# Do pilot trials reliably predict recruitment and retention rates for full trial? A review of HTA funded trials

**DOI:** 10.1186/1745-6215-16-S2-P14

**Published:** 2015-11-16

**Authors:** Amy Whitehead, Cindy Cooper, Steve Julious, Edward Pottrill

**Affiliations:** 1 University of Sheffield, Sheffield, UK

## Background

Pilot trials are recommended for testing the feasibility of full trials. However, there is little evidence that they actually predict progress in the full trial. We compared randomisation and attrition rates for HTA funded RCTs with their pilots.

## Methods

RCTs for which there was an external pilot trial were identified from HTA monographs published between 2004 and 2013. Data were extracted from published papers, protocols and monographs. Bland-Altman plots and descriptive statistics were used to investigate the agreement of randomisation and attrition rates between the full and pilot trials.

## Results

Of 561 available HTA monographs 40 were RCTs with pilot trials and 17 met criteria for a pilot trial and had sufficient data. The mean attrition and randomisation rates were 17.66% and 0.42 respectively in the pilot and 16.44% and 0.59 in the main trials.

There was minimal bias in the pilot trial when predicting the main trial attrition and randomisation rate. However, the variation was large: he mean difference in the attrition rate between the pilot and the main trial was -1.22% with limits of agreement of -25.48 to 23.05%. The majority of trials were within +/ 10%. The limits of agreement for randomisation rates were -3.00 to 1.75.

## Conclusions

Results from external pilot trials to estimate randomisation and attrition rates should be used with caution as comparison with the full trial demonstrates high variability. We suggest using internal pilot trials wherever appropriate.

